# Editorial: Graph learning for brain imaging

**DOI:** 10.3389/fnins.2022.1001818

**Published:** 2022-08-22

**Authors:** Feng Liu, Yu Zhang, Islem Rekik, Yehia Massoud, Jordi Solé-Casals

**Affiliations:** ^1^School of Systems and Enterprises, Stevens Institute of Technology, Hoboken, NJ, United States; ^2^Department of Bioengineering, Lehigh University, Bethlehem, PA, United States; ^3^Department of Electrical and Computer Engineering, Lehigh University, Bethlehem, PA, United States; ^4^BASIRA Lab, Faculty of Computer and Informatics Engineering, Istanbul Technical University, Istanbul, Turkey; ^5^Department of Electrical and Computer Engineering, King Abdullah University of Science and Technology, Makkah, Saudi Arabia; ^6^Department of Engineering, University of Vic-Central University of Catalonia, Vic, Spain; ^7^Department of Psychiatry, University of Cambridge, Cambridge, United Kingdom

**Keywords:** graph learning, brain networks, deep learning, brain imaging, graph neural networks (GNN), multimodality

Unprecedented collections of large-scale brain imaging data, such as MRI, PET, fMRI, M/EEG, DTI, etc. provide a unique opportunity to deepen our understanding of the brain working mechanisms, improve prognostic predictions for mental disorders, and tailor personalized treatment plans for brain diseases. Recent advances in machine learning and large-scale brain imaging data collection, storage, and sharing lead to a series of novel interdisciplinary approaches in the fields of computational neuroscience, signal processing, deep learning, brain imaging, cognitive science, and computational psychiatry, among which graph learning provides a valuable means to address important questions in brain imaging.

Graph learning refers to designing effective machine learning and deep learning methods to extract important information from graphs or exploiting the graph structure in the data to guide knowledge discovery. Given the complex data structure in different imaging modalities as well as the networked organizational structure of the human brain, novel learning methods based on graphs inferred from imaging data, graph regularizations for the data, and graph embedding of the recorded data, have shown great promise in modeling the interactions of multiple brain regions, information fusion among networks derived from different brain imaging modalities, latent space modeling of the high dimensional brain networks, and quantifying topological neurobiomarkers. This Research Topic synergizes the state-of-the-art discoveries in terms of new computational brain imaging models and insights into brain mechanisms through the lens of brain networks and graph learning.

We accepted 10 manuscripts recommended by the reviewers after evaluating the novelty and quality of the contributions. In order to introduce these works in more detail, we highlight three domains in this Editorial that emerge from the 10 contributions to this Research Topic.

(1) *Leveraging graph theory and network analysis to identify the biomarkers of brain disorders*. Specifically, Cui et al. used graph theory analysis based on fMRI to investigate alterations of brain functional networks in profound bilateral congenital sensorineural hearing loss (SNHL) in infants, and this study also provided novel insights into functional network alterations in the early stage of profound bilateral congenital SNHL. Zhu et al. explored the aberrant functional connectivity of sensory motor networks in BD-I (bipolar disorder type I) patients and its associations with executive dysfunction. The authors found a significant relationship between the abnormal intranetwork and internetwork functional connectivity values, clinical symptoms and executive function, which provides new information for exploring the neural physiopathology of executive dysfunction in BD-I patients. Chen Y. et al. proposed an invertible dynamic Graph Convolutional Network (GCN) model to identify Autism Spectrum Disorder (ASD) and investigate the alterations of connectivity patterns associated with the disorder. Their proposed method achieves superior classification performance, which provides an interpretable deep learning model for brain connectivity analysis and is of great potential in studying other brain-related disorders.
(2) *Using new machine learning frameworks to understand the functional and structural brain maps, and an integration of both functional and structural brain networks*. In this category, Jon Albers et al. presented a novel approach for quantifying the relationship between brain function and structure and the integration of these in terms of processing units. Their proposed framework naturally can be extended to a general multimodal modeling framework. Eschenburg et al. proposed a cortical segmentation method that, given resting-state connectivity features readily computed during conventional MRI pre-processing and a set of corresponding training labels, can generate cortical parcellations for new MRI data. They found that, in all cases, graph neural networks consistently and significantly outperformed a baseline neural network. Qiu et al. proposed an individualized cortical parcellation based on graph neural networks to learn the reliable functional characteristics of each brain parcel on a large fMRI dataset and to infer the areal probability of each vertex on unseen subjects. This study provides new avenues for precise mapping of cortical areas onto individual brains, and shows potential applications in locating personalized functional areas in the diagnosis and treatment of neurological disorders.
(3) *Methodology oriented papers for data augmentation, multimodal fusion, and graph signal processing*. For example, Zhang et al. proposed a novel approach to generate a fused cognitive network with the optimal performance in discriminating cognitive states by using graph learning. Their findings suggest that the fused cognitive network provides the potential to develop new mind decoding approaches. Chen X. et al. proposed to use a data augmentation method by adding artificial samples generated using graph empirical mode decomposition, which can improve the average classification performance. Furthermore, their augmentation method can be extended to other similar small datasets. Jiao et al. proposed to use the low-frequency components to approximate the extended source activation after graph Fourier transform (GFT) and built a bidirectional long-short term memory (BiLSTM) neural network to solve the Electrophysiological source imaging problem. Chan et al. proposed a new framework called Joining Omics and Imaging Networks *via* Graph Convolutional Layers and Attention (JOIN-GCLA), which consists of multiple graph convolution layers and an attention mechanism to combine multi-modal imaging data and multi-omics datasets for the prediction of PD. The JOIN-GCLA architecture makes it possible to analyze multi-modal imaging data along with multi-omics datasets.

These collected articles have made outstanding contributions to the field of brain science and brain imaging. The research can make a broader impact on the brain disorder diagnostic and prognostic analysis by using network theory, deep learning, and graph signal processing.

## Author contributions

All authors listed have made a substantial, direct, and intellectual contribution to the work and approved it for publication.

## Conflict of interest

The authors declare that the research was conducted in the absence of any commercial or financial relationships that could be construed as a potential conflict of interest.

## Publisher's note

All claims expressed in this article are solely those of the authors and do not necessarily represent those of their affiliated organizations, or those of the publisher, the editors and the reviewers. Any product that may be evaluated in this article, or claim that may be made by its manufacturer, is not guaranteed or endorsed by the publisher.

